# Particulate air pollution on cardiovascular mortality in the tropics: impact on the elderly

**DOI:** 10.1186/s12940-019-0476-4

**Published:** 2019-04-18

**Authors:** Jonathan Yap, Yixiang Ng, Khung Keong Yeo, Anders Sahlén, Carolyn Su Ping Lam, Vernon Lee, Stefan Ma

**Affiliations:** 1 Department of Cardiology, National Heart Centre Singapore, 5 Hospital Drive, Singapore, 169609 Singapore; 2 Public Health Group, Ministry of Health, Singapore, Singapore; 3 Duke-NUS Graduate Medical School, Singapore, Singapore; 4 Karolinska Institutet, Stockholm, Sweden; 5 Saw Swee Hock School of Public Health, National University of Singapore, Singapore, Singapore

**Keywords:** Air pollution, Cardiovascular mortality, Tropics, Elderly

## Abstract

**Background:**

Air pollution has a significant health impact. Most data originate from temperate regions. We aim to study the health impact of air pollution, particularly among the elderly, in a tropical region.

**Methods:**

A daily time-series analysis was performed to estimate excess risk (ER) of various air pollutants on daily death counts amongst the general population in Singapore from 2001 to 2013. Air pollutants included particulate matters smaller than 10 μm, and 2.5 μm (PM_10_, PM_2.5_), carbon monoxide (CO), nitrogen dioxide (NO_2_), ozone (O_3_) and sulphur dioxide (SO_2_). The studied outcomes were non-accidental and cardiovascular mortality. Single-day lag and distributed lag models were studied and adjusted for confounders.

**Results:**

In single-day lag models, a 10 μg/m^3^ increase in particulate matter was associated with significant increases in non-accidental (PM_10_ ER: 0.627%; 95% confidence interval (CI): 0.260–0.995% and PM_2.5_ ER: 0.660%; 95% CI: 0.204–1.118%) and cardiovascular mortality (PM_10_ ER: 0.897; 95% CI: 0.283–1.516 and PM_2.5_ ER: 0.883%; 95% CI: 0.121–1.621%). This was significant in the elderly ≥ 65 years but not in those < 65 years and were seen in the acute phase of lag 0-5 days. Effects by other pollutants were minimal. For cardiovascular mortality, the effects turned protective at a cumulative lag of 30 days in the elderly and could due to “harvesting”.

**Conclusions:**

These first contemporary population-based data from an equatorial country with tropical climate show that exposure to particulate air pollution was significantly associated with non-accidental mortality and cardiovascular mortality, especially in the elderly.

**Electronic supplementary material:**

The online version of this article (10.1186/s12940-019-0476-4) contains supplementary material, which is available to authorized users.

## Background

Air pollution has a significant health impact on mortality and morbidity worldwide, resulting in an estimated 4.2 million deaths and 103.1 million disability–adjusted life-years lost [1]. Air pollution has wide-ranging systemic effects on the human body, impacting both the respiratory, and cardiovascular systems via multiple mechanisms including oxidative stress, inflammation and endothelial dysfunction [2].

Many prospective cohort and daily time-series studies published globally have consistently demonstrated the negative associations between long and short-term exposure to air pollution and human health [3–9]. These studies did not only establish associations between ambient particulate matters and respiratory health, but also on cardiovascular health, with the elderly being an especially susceptible group [10–12]. However, the vast majority of these studies were conducted in temperate regions [3–9] rather than in the tropics. As seasonal variations and temperature changes have been shown to impact the relationship of air pollution on health outcomes [13–15], we aim to study the impact of air pollution on non-accidental and cardiovascular mortality in the general population, as well as the elderly, in an equatorial country with tropical climate and no seasons.

## Methods

### Data sources

Singapore is an Asian city-state, situated near the equator with a tropical climate, comprising 5.40 million of people. Besides being exposed to daily ambient air pollution generated from domestic sources, Singapore is also exposed almost yearly to haze episodes of about a month long duration, whereby smoke from regional forest fires especially during the dry seasons is blown by winds from neighbouring countries [16].

Air pollutant and meteorological data are comprehensively collected in Singapore. Daily average of 24-h concentrations for particulate matters smaller than 10 μm and 2.5 μm (PM_10_ and PM_2.5_), 8-h carbon monoxide (CO), 24-h nitrogen dioxide (NO_2_), 8-h ozone (O_3_) and 24-h sulphur dioxide (SO_2_) from the years of 2001 to 2013 were obtained from the National Environment Agency (NEA) Singapore. During this 13-year study period, air pollution in Singapore was monitored in air monitoring stations located at various sites around Singapore. Four stations located at road-sides were excluded from the study because they did not reflect the daily exposure to air pollutants amongst the general population. Our final analysis included data from 18 studied stations and data completeness for each studied station was assessed by calculating the proportion of days on which data was collected out of the number of days in operation. Aggregated daily air pollutant concentrations were calculated following the Air Pollution on Health: European Approach (APHEA) protocol with added modifications [13]. In summary, imputed annual mean concentrations for each station were subtracted from the station-specific daily concentrations of the same year to generate a set of ‘centred’ values. Using the centred values, the arithmetic mean was calculated across all stations by day and the average of the imputed annual mean concentrations were then added back to derive the daily values used in the analysis. Average meteorological values were derived from daily means of dry bulb temperature and relative humidity from 5 stations which selected based on the shortest proximity to the air monitoring stations provided by NEA’s Meteorological Service division.

Mortality data from the Registry of Births & Deaths were extracted to calculate aggregated daily counts of all non-accidental deaths (International Classification of Diseases (ICD)-9000–799 from the period of 2001–2012 & ICD-10 A00-R99 for the year of 2013) and cardiovascular deaths (ICD-9390–459 & ICD-10 I00-I99) by age groups (all-age, < 65 and ≥ 65 years) over the 13 years study period. A separate analysis was also conducted looking at the subset of subjects ≥ 80 years. Ethics approval was obtained from the SingHealth Centralised Institutional Review Board.

### Statistical analysis

A quasi-Poisson generalized additive model was used for the analysis [17]. Single-day lag models and distributed lag models (DLMs) were respectively built to analyse the lagged day effects of the different air pollutants first on all ages combined non-accidental and cardiovascular mortality [18–20] respectively. Analyses of PM_10_ and PM_2.5_ effects stratified by two age groups (< 65 and ≥ 65 years) were then repeated using the DLMs to further study age-specific effects.

Separate core models were firstly built (for each age group and mortality type) without adding pollutant variable to explain the variations, as much as possible, due to long term trends and other potential time-varying confounding factors:

$$ Log\ E\left[(Y)\right]=\alpha +s(T)+{\beta}_1 DOW+{\beta}_2 SARS+{\beta}_3 FLU+{\beta}_4 PH+{\beta}_5 afterPH+s(DBT)+s(RH) $$

Where *Log* is the natural logarithm, *Y* is the daily counts of non-accidental or cardiovascular deaths, DOW refers to the day of week and *s*(*T*), *s*(*DBT*) and *s*(*RH*) refer to the penalised cubic regression smoothers for long term trends, dry bulb temperature (°C) and relative humidity (%) respectively. 1 to 3 days lag were tested for variables *DBT* and *RH*. Selection of lag-day variables and smoothing spline parameters were based on models giving the lowest quasi-Akaike’s Information Criterion [21]. Dummy variables of *SARS* and *FLU* are used to control for impact due to the periods of 2003 severe acute respiratory syndrome (SARS) and of the 2009 influenza A(H1N1) pandemics respectively while *PH* and *afterPH* are terms for public holidays and the day following public holidays.

After the core model was established and chosen for each mortality outcome, variables depicting the various air pollutants were added for the single-day lag models. Two-pollutant models were further constructed for pollutants with significant effects in the single pollutant model. For the DLMs, basis functions were applied with the lag-response relationship defined a priori using a 3rd degree polynomial. Polynomials had been used in previous air pollution studies to analyse the lag-response associations [22–24]. A 30-day lag structure, signifying a medium-term period of about a month, was used to observe cumulative effects and mortality displacement if any. As a sensitivity analysis, cumulative lag-response charts generated with DLMs using 2nd and 4th degrees polynomials are shown in the Additional file 1: Figures S1 to S4. Percentage changes in mortality risk, or excess risks (ERs), associated with a 0.1 mg/m^3^ for CO and 10 μg/m^3^ for other pollutants were calculated using (RR-1) × 100%, where RR denotes relative risk estimated from the regression coefficient of air pollutant variable of the models. Statistical significance was assessed using 95% confidence intervals (CI). Residual autocorrelation (ACF) and partial autocorrelation (PACF) charts for the core models were performed to assess model fit and are shown in Additional file 1: Figure S5. Data collection process might be interrupted for certain stations over the study period due to site relocation or site closure, and hence annual mean pollutant concentrations used for centring the data were imputed using spatial interpolation with the “fields” package version 8.3–6 in the R statistical software [25]. All statistical analyses, including plotting of lag-response relationships and residual diagnostics to assess model fit, were carried out using the “mgcv” and “dlnm” packages in R version 3.3.1 [17, 19, 26].

## Results

Mean daily concentrations of PM_10_ & PM_2.5_ were 29.4 μg/m^3^ and 20.0 μg/m^3^ respectively, and reached as high as 336.6 μg/m^3^ and 275.8 μg/m^3^ during the haze episode in 2013. CO concentration also reached a high of 3.6 mg/m^3^ during the same episode. Given Singapore’s tropical climate, daily mean of dry bulb temperatures ranged narrowly from 23.4 °C to 30.9 °C while daily mean of relative humidity ranged from 63.6 to 98.1%. Mean daily counts of non-accidental and cardiovascular mortality for all-ages were 43.5 and 15.4 respectively while figures of 30.6 for non-accidental mortality and 11.2 for cardiovascular mortality were recorded in the elderly aged 65 years old and above (Table 1). The correlation between air pollutant and meteorological data is found in the Additional file 1: Table S1. There is a very high linear correlation between levels of PM_10_ and PM_2.5_ (*r* = 0.923).

**Table 1:** Summary statistics of daily air pollutant concentrations, meteorological data and mortality.2001–2013

	No.(days)	Mean	SD	Percentile				
				Min	10th	50th	90th	Max
Air Pollutants								
PM_10_ (μg/m^3^)	4748	29.4	12.9	7.6	19.1	27.6	39.9	336.6
PM_2.5_ (μg/m^3^)	4748	20.0	10.6	6.0	11.9	18.1	28.9	275.8
CO (mg/m^3^)	4748	0.6	0.2	0.1	0.3	0.5	0.8	3.6
NO_2_ (μg/m^3^)	4748	25.0	6.8	7.4	16.8	24.4	33.7	60.7
O_3_ (μg/m^3^)	4747^a^	37.7	15.1	5.4	20.1	35.8	56.9	125.5
SO_2_ (μg/m^3^)	4748	13.2	7.6	0.5	4.2	12.3	23.2	60.8
Meteorological variables								
DBT (°C)	4748	27.8	1.1	23.4	26.3	27.8	29.2	30.9
Relative humidity (%)	4748	81.4	5.3	63.6	74.6	81.2	88.5	98.1
Daily non-accidental mortality								
All ages	4748	43.5	7.7	21	34.0	43.0	54.0	74
< 65 years	4748	12.9	3.6	2	8.0	13.0	18.0	27
> 65 years	4748	30.6	6.5	13	23.0	30.0	39.0	57
Daily cardiovascular mortality								
All ages	4748	15.4	4.1	4	10.0	15.0	21.0	33
< 65 years (No.)	4748	4.3	2.1	0	2.0	4.0	7.0	16
> 65 years (No.)	4748	11.2	3.4	2	7.0	11.0	16.0	26

^a^No data for ozone on 11/2/2012

Completeness of the air pollutant data ranged from 58.2 to 100.0%. For each pollutant, there were 10 to 17 stations that collected data on more than 80% of the days that they were in operation during the study period. There was only one day in 2012 when data for zone was not available from any of the stations.

### Air pollution and mortality

The low residual ACF and PACF values (Additional file 1: Figure S5) showed that the core models were able to explain the temporal trends of the data adequately. Significant effects were observed for PM_10_ & PM_2.5_ in both the single-day and distributed lag models. In the single-day lag models, the highest ER associated with a 10 μg/m^3^ increase occurred in the 3rd day after exposure for both non-accidental (PM_10_ ER: 0.627, 95% CI: 0.260–0.995% and PM_2.5_ ER: 0.660%; 95% CI: 0.204–1.118%) and cardiovascular mortality (PM_10_ ER: 0.897%; 95% CI: 0.283–1.516% and PM_2.5_ ER: 0.883%; 95% CI: 0.121–1.621%). The estimated ERs generally remained significant for PM_10_ and PM_2.5_ even after controlling for additional pollutants (Additional file 1: Table S2a,b).

O_3_ also exhibited a significant effect at day 1 lag for non-accidental mortality (ER: 0.354%; 95% CI: 0.011–0.698%). However, this effect was insignificant after adjusting for PM_10_ and PM_2.5_ (Additional file 1: Table S2c). Effects by other pollutants were observed to be minimal or insignificant (Table 2).

**Table 2:** Single-day lag models for association between air pollutant levels and mortality^a^

	Percentage change (95% confidence interval)			
	Lag 0	Lag 1	Lag 2	Lag 3
Non-accidental mortality				
PM_10_	**0.485 (0.109, 0.863)**	**0.487 (0.114, 0.862)**	**0.481 (0.111, 0.852)**	**0.627 (0.260, 0.995)**
PM_2.5_	**0.593 (0.129, 1.058)**	**0.518 (0.056, 0.981)**	**0.504 (0.047, 0.964)**	**0.660 (0.204, 1.118)**
CO	0.064 (−0.237, 0.366)	0.133 (− 0.166, 0.433)	0.240 (− 0.059, 0.539)	0.219 (− 0.077, 0.514)
NO_2_	− 0.124 (− 0.985, 0.744)	− 0.025 (− 0.867, 0.824)	− 0.082 (− 0.947, 0.791)	0.042 (− 0.800, 0.890)
O_3_	0.015 (− 0.372, 0.403)	**0.354 (0.011, 0.698)**	0.037 (− 0.356, 0.432)	0.219 (− 0.117, 0.557)
SO_2_	− 0.218 (− 1.044, 0.616)	0.221 (− 0.603, 1.053)	0.679 (− 0.147, 1.512)	−0.390 (− 1.208, 0.435)
Cardiovascular mortality				
PM_10_	0.553 (− 0.074, 1.185)	**0.633 (0.007, 1.262)**	0.610 (− 0.010, 1.234)	**0.897 (0.283, 1.516)**
PM_2.5_	0.632 (−0.142, 1.412)	0.603 (−0.172, 1.385)	0.541 (− 0.229, 1.318)	**0.883 (0.121, 1.651)**
CO	0.167 (−0.328, 0.665)	0.071 (−0.418, 0.561)	0.135 (− 0.356, 0.629)	0.362 (− 0.132, 0.858)
NO_2_	− 0.344 (− 1.707, 1.038)	−0.151 (− 1.486, 1.203)	−0.897 (− 2.217, 0.441)	−0.352 (− 1.726, 1.041)
O_3_	0.500 (− 0.134, 1.137)	0.292 (− 0.266, 0.854)	0.105 (− 0.454, 0.667)	0.489 (− 0.154, 1.137)
SO_2_	0.223 (− 1.114, 1.577)	−0.325 (− 1.647, 1.014)	0.857 (− 0.479, 2.211)	−0.197 (− 1.519, 1.143)

^a^Per 0.1 mg/m^3^ increase in CO and per 10 μg/m^3^ increase for other pollutants

Results highlighted in bold are statistically significant

**Fig. 1:**
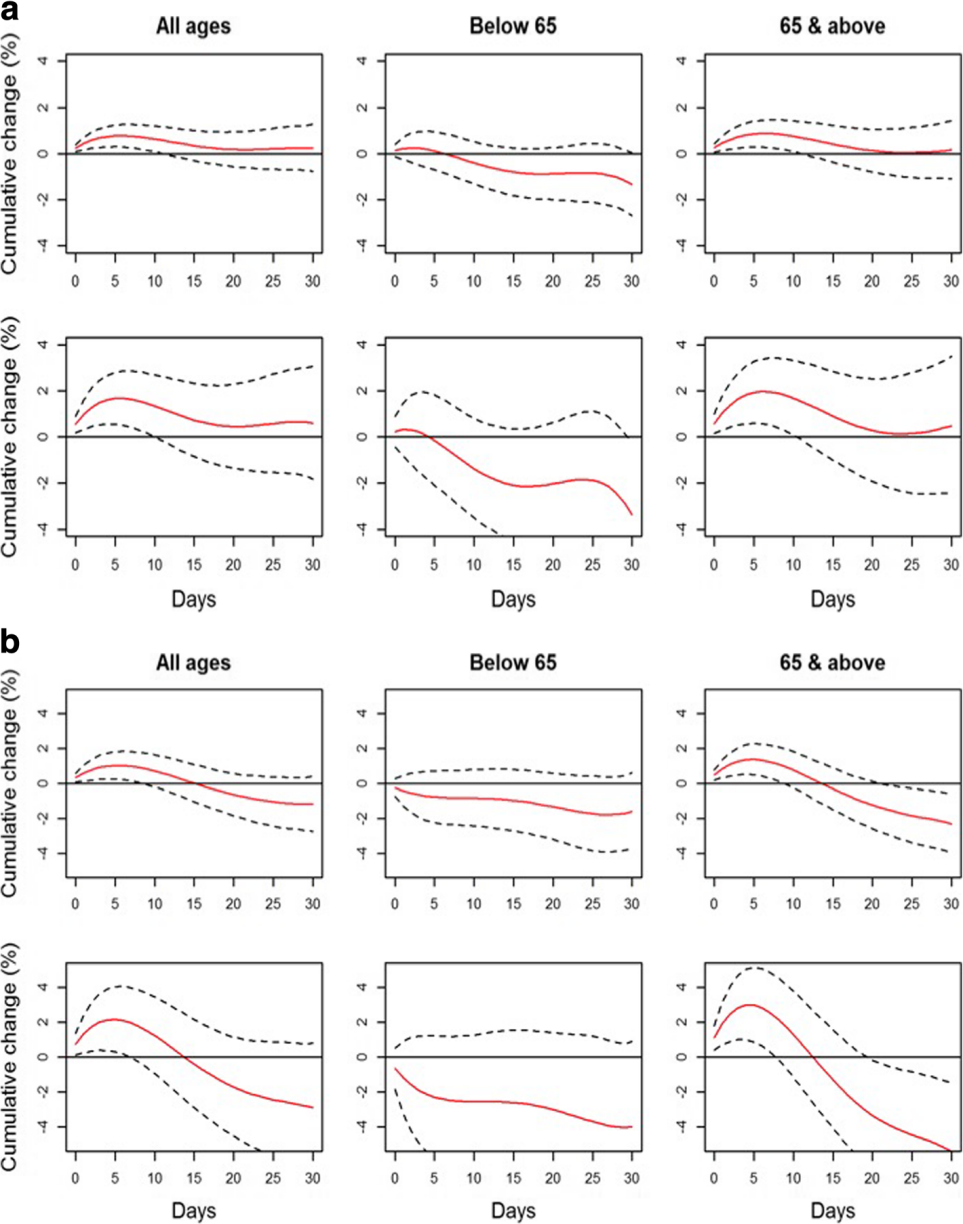
**a** Cumulative percent change (%) in non-accidental mortality for 10 μg/m^3^ pollutant concentration increase in PM_10_ (top row) and PM_2.5_ (bottom row). **b** Cumulative percent change (%) in cardiovascular mortality for 10 μg/m^3^ pollutant concentration increase in PM_10_ (top row) and PM_2.5_ (bottom row)

Results from the distributed lag models are presented in Table 3 and Fig. 1. Significant cumulative effects were observed over days 0–5 but not over subsequent longer periods. Cumulative ERs over days 0–5 for PM_10_ & PM_2.5_ were estimated to be 0.700% (95% CI: 0.276–1.126%) & 0.829% (0.276–1.386%) respectively for non-accidental [30] mortality, and 0.921% (0.218–1.629%) & 1.073% (0.157–1.998%) respectively for cardiovascular mortality. Sensitivity analysis using 2nd or 4th degree polynomials showed similar results (Additional file 1: Figures S1-S4).

**Table 3:** Distributed lag models for association between air pollutant levels and mortality^a^

	Percentage change (95% confidence interval)		
	Lags 0–5	Lags 0–15	Lags 0–30
Non-accidental mortality			
PM_10_	**0.700 (0.276, 1.126)**	0.316 (−0.277, 0.912)	0.220 (−0.700, 1.148)
PM_2.5_	**0.829 (0.276, 1.386)**	0.362 (−0.419, 1.150)	0.297 (−0.904, 1.512)
CO	0.260 (−0.051, 0.572)	0.074 (−0.368, 0.518)	0.660 (− 0.015, 1.339)
NO_2_	−0.622 (−1.713, 0.480)	−1.560 (−3.119, 0.024)	−0.106 (−2.239, 2.073)
O_3_	0.123 (−0.373, 0.622)	− 0.628 (− 1.305, 0.054)	− 1.184 (− 2.200, − 0.159)
SO_2_	−0.125 (− 1.380, 1.146)	0.120 (− 1.715, 1.989)	1.048 (− 1.514, 3.677)
Cardiovascular mortality			
PM_10_	**0.921 (0.218, 1.629)**	0.011 (−0.948, 0.989)	− 1.078 (− 2.489, 0.354)
PM_2.5_	**1.073 (0.157, 1.998)**	−0.196 (− 1.451, 1.074)	− 1.450 (− 3.263, 0.396)
CO	0.278 (− 0.232, 0.791)	− 0.151 (− 0.854, 0.557)	0.110 (− 0.922, 1.152)
NO_2_	−1.345 (− 3.034, 0.375)	−3.112 (−5.365, − 0.805)	−1.685 (−4.498, 1.211)
O_3_	0.435 (− 0.350, 1.227)	−0.785 (− 1.809, 0.249)	−2.165 (− 3.586, − 0.722)
SO_2_	−0.095 (− 2.098, 1.948)	−0.963 (− 3.747, 1.901)	−1.032 (− 4.653, 2.727)

^a^Per 0.1 mg/m^3^ increase in CO and per 10 μg/m^3^ increase for other pollutants

Results highlighted in bold are statistically significant

### Impact by age

Cumulative effects were significant in the elderly aged 65 years old and above, with every 10 μg/m^3^ increase in pollutant concentration resulting in a 0.771% (95% CI: 0.265–1.279%) and a 0.955% (0.297–1.618%) increase over days 0–5 in non-accidental mortality risk for PM_10_ & PM_2.5_ respectively. Cardiovascular mortality in turn showed a 1.236% (0.436–2.042%) and a 1.478% (0.437–2.530%) increase in risk of PM_10_ & PM_2.5_ over days 0–5 (Table 4).

**Table 4:** Distributed lag models for association between air pollutant levels and mortality by age group^a^

		Percentage change (95% confidence interval)		
		Lags 0–5	Lags 0–15	Lags 0–30
Non Accidental Mortality				
< 65 years	PM_10_	0.105 (− 0.641, 0.856)	− 0.726 (− 1.659, 0.216)	− 1.214 (− 2.442, 0.029)
	PM_2.5_	−0.073 (− 1.039, 0.903)	− 1.045 (− 2.246, 0.171)	**−1.694 (− 3.220, − 0.143)**
> 65 years	PM_10_	**0.771 (0.265, 1.279)**	0.376 (− 0.345, 1.102)	0.146 (− 0.980, 1.284)
	PM_2.5_	**0.955 (0.297, 1.618)**	0.447 (− 0.501, 1.405)	0.241 (− 1.229, 1.733)
Cardiovascular Mortality				
< 65 years	PM_10_	− 0.706 (− 2.038, 0.645)	− 0.885 (− 2.466, 0.721)	−1.456 (− 3.411, 0.539)
	PM_2.5_	−1.145 (−2.872, 0.613)	− 1.302 (− 3.332, 0.771)	−2.015 (− 4.417, 0.447)
> 65 years	PM_10_	**1.236 (0.436, 2.042)**	− 0.288 (− 1.366, 0.801)	**−2.085 (− 3.614, − 0.532)**
	PM_2.5_	**1.478 (0.437, 2.530)**	−0.655 (− 2.060, 0.771)	**−2.721 (− 4.667, − 0.736)**

^a^Per 10 μg/m^3^ increase in PM_10_ and PM_2.5_

Results highlighted in bold are statistically significant

No significant effects were seen over days 0–15, but interestingly significant negative cumulative effects were seen for cardiovascular mortality over days 0–30 in the elderly group (PM_10_ ER: -2.085%; 95% CI: -3.614% – -0.532% and PM_2.5_ ER: -2.721%; 95% CI: -4.667% – -0.736%). A similar negative effect was also seen for the younger age group for non-accidental mortality over days 0–30 for PM_2.5_ (ER:-1.694%; 95% CI: -3.220% – -0.143%). Sensitivity analysis using up to 2nd or 4th degree of polynomials showed similar results (Additional file 1: Figures S1-S4).

In a separate analysis looking at the very elderly aged 80 years and above, cumulative effects were significant, with every 10 μg/m^3^ increase in pollutant concentration resulting in a 0.749% (95% CI: 0.095–1.408%) and a 0.955% (0.105–1.812%) increase over days 0–5 in non-accidental mortality risk for PM_10_ & PM_2.5_ respectively (data not shown). There was a trend towards increased cardiovascular mortality at days 0–5, although this association was not significant. Significant negative cumulative effects were seen for cardiovascular mortality over days 0–30 in this very elderly group for PM_10_ (ER: -2.060%; 95% CI: -3.987% – -0.094%) and a similar non-signficant trend for PM_2.5_.

### Lag-response association

Graphs showing non-cumulative percentage change in mortality over days 0–30 are shown in the Additional file 1: Figure S6, S7. The graphs for the all-age group and the ≥ 65 years age group showed that impact on mortality was mostly observed in the immediate days (days 0–5) after exposure. After this initial period, significant protective effects were actually observed in both non-accidental & cardiovascular mortality. However, while non-accidental mortality showed effects approaching the null around days 15–20, cardiovascular mortality only showed a rebound around days 20–25, thus indicating a slower approach.

## Discussion

In this daily time series analysis performed in an equatorial Asian city-state with tropical climate, we found significant associations between particulate air pollutants (PM_2.5_, PM_10_) and non-accidental mortality, as well as cardiovascular mortality, which corroborated with other previous studies conducted in temperate countries, but our estimated effect sizes are closer to the lower end of their estimates. Greater effects were especially found in the elderly. The impacts of other pollutants were generally insignificant however.

The strongest evidence for the impact of air pollutants on mortality lie in the particulate air pollutants. In a recent meta-analysis, a 10 μg/m^3^ increment in PM_2.5_ was associated with a 1.04% increase in mortality with substantial regional variations (ranging from 0.25 to 2.08%) [27]. In a study from 20 cities in the United States, a 10 μg/m^3^ increased in PM_10_ was associated with significant increase of 0.51% in overall mortality, with no impact from the other air pollutants [7]. Another study from 38 cities in China similarly showed a 10 μg/m^3^ change in PM_10_ concentrations was associated with a 0.44% increase in daily number of deaths [3]. In Europe, PM_2.5_ was significantly associated with overall mortality while NO_2_ was not [5]. The ESCALA study (Estudio de Salud y Contaminación del Aire en Latinoamérica) which included 9 Latin American cities in the tropics (albeit non-equatorial) found a similar significant association between PM_10_ and mortality [28]. In our study, we found an increase of 0.63 and 0.66% in overall mortality with every 10 μg/m^3^ increase in PM_10_ and PM_2.5_ respectively from the single-day lag model. No significant impact on overall mortality was found from the other air pollutants. The lack of impact from other air pollutants despite some findings from other studies may in part be explained by the lower concentrations of these air pollutants in our study. For example, the NO_2_ concentrations in our study were about half that of several published studies [22, 29] which found significant associations between NO_2_ and mortality. Similarly, significant lower concentrations of O_3_ was noted in our study compared to others [28].

Since the hypothesis was first postulated by Seaton et al. (1995), that ultra-fine particulates, less than 0.3 μm in aerodynamic diameter, may provoke more alveolar inflammation causing exacerbation of existing lung disease and increased blood coagulability than larger respired particles, the impact of air pollution on cardiovascular health has received great attention. In addition, air pollutants result in increase in risk of cardiovascular events via various pathways including oxidative stress, systemic inflammation, endothelial dysfunction, atherothrombosis, and arrhythmogenesis [2, 29, 30]. Increased levels of air pollutants have been shown to be associated with progression in coronary calcification on computed tomography scans [8]. The same meta-analysis mentioned in the previous paragraph found a 0.84% increase in cardiovascular mortality with a 10 μg/m^3^ in PM_2.5_ [27]. The ESCALA study from tropical Latin American also found a significant association between PM_10_ and cardiovascular events [28]. In study of 8 cities in China, a 10 μg/m^3^ in PM_10_ was associated with 0.36% increase in coronary heart disease mortality, with significant associations also found for NO_2_ and SO_2_ [31]. A study from Korea found that exposure to PM_2.5_, PM_10_, CO, SO_2_, and NO_2_, but not O_3_, increased the risks of cardiovascular events and mortality [6].

In our analysis of the single-day lag model, we found an increase of 0.90 and 0.88% in cardiovascular mortality with every 10 μg/m^3^ increase in PM_10_ and PM_2.5_ respectively. Interestingly, in the single-day lag model, the earliest significant effects on overall mortality were seen on the same day as exposure while the effects on cardiovascular mortality were primarily seen on lag day 3. Although both lags are fairly acute, this may indicate a relatively more delayed effect of air pollution on the cardiovascular system compared to the respiratory system (the respiratory system being in direct contact with the inhaled pollutants). Furthermore, although the pro-ischemic and pro-thrombotic effects of pollution may occur within hours of exposure [32], it may take longer (eg. days) for cardiovascular mortality to occur. A similar finding was demonstrated by Costa et al., whereby the impact of air pollution on cardiovascular mortality was evident only on lag day 3 while the impact on respiratory mortality was more immediate [22]. We did not find an impact of other pollutants on cardiovascular mortality.

The elderly is an increasingly vulnerable group and sensitive to the effects of particulate matter. The elderly tends to be frailer, have more co-morbidities and less physiological reserves. In the Chinese study, the effect of PM_10_ on overall mortality in those less than 60 years old was insignificant but became significant in those aged ≥ 60 years old (0.57% per 10μg/m^3^ increase) [3]. In the Netherlands, PM_10_ caused increased overall mortality in all age groups but only caused significant circulatory disease mortality in those aged > 65 years old [4]. Multiple other studies have also demonstrated the ill effects from air pollution in the elderly age group. In our study, we found similar significant associations with overall and cardiovascular mortality with PM_10_ and PM_2.5_ in the elderly aged ≥65 years but not in those < 65 years. The significant effects on mortality were seen primarily in the acute phase of lag 0-5 days. Interestingly, for cardiovascular mortality, the effects turned protective at a cumulative lag of 30 days in the elderly. This suggests that the number of cardiovascular deaths was lower as compared to another 30-day window period where no exposure to particulate matter had taken place. This could be attributed to the “mortality displacement” or “harvesting” effect [23, 33]. In the elderly population, high level of short term exposure to air pollution may cause greater mortality, advancing deaths and depleting the ‘at-risk’ group early on, resulting in a follow-on period with a mortality rate that is lower than expected [23, 33]. In a study on air pollution and myocardial infarction, higher levels of PM10 were associated with short-term increased risk of MI, but later reductions in risk suggest that air pollution may be associated with bringing events forward in time (“short-term displacement”) [34]. Another reason for the apparent lower number of deaths later on might be the subsequent protective measures (e.g. wearing masks, staying indoors) that alerted individuals or organizations put in place during periods of high air pollution levels. This may in part explain the lower non-accidental mortality in the younger age group at cumulative lag of 0–30 days.

The vast majority of studies on the impact of air pollution were performed in temperate regions. The meta-analysis used data mainly from European, American and Western Pacific regions, with only one study on hospital admissions that included data from south-east Asia [27]. The Global Burden of Diseases Study included data from tropical regions but air pollution burden was largely estimated from satellite data and also extrapolated and applied risk estimates of air pollution from other studies for places where such estimates were not available [1]. Moreover, studies have shown that seasonal changes, temperature and humidity variation may impact the relationships between air pollution effect and health outcomes [13–15], emphasizing the importance of validating these findings in the tropics, where the climate is starkly different. In the APHEA study, there was higher effects of PM_10_ on daily mortality in cities with a higher temperatures [13] and similarly in Korea, hotter temperatures accentuated the effect of PM_10_ on mortality [14]. In our study, we found similar significant findings on the negative impact of particulate air pollution on overall and cardiovascular mortality. The estimates from our study, although not directly comparable to other studies, appear to be on the lower end of the spectrum.

### Limitations

There are several limitations. Firstly, due to the time-series analysis nature of the study, unmeasured clinical variables (eg. comorbidities), which could affect the actual estimates of air pollution on mortality, could not be fully accounted for despite our best efforts in controlling for potential confounding factors in the models. Our models also did not adjust for the transboundary haze episodes as we wanted to analyse the general exposure of the public to air pollutants, and not to distinguish between domestic and transboundary air pollution. Secondly, the impact of indoor air pollution was not assessed as this data was not readily available, but we believe such effect would be minimal as residents in Singapore do not burn coals for cooking in the houses. Thirdly, a 3rd degree of polynomials was used to define the lag-response relationship over a period of 30 days. A different parametric shape or a different degree parameter would result in different ER estimates. However, a sensitivity analysis using up to 2nd or 4th degree of polynomials showed that these differences were minute and did not affect our final interpretation. A fourth limitation is that we did not explore the inclusion of different concentration-response functions to describe the exposure-response relationship [35]. In our analysis, we assumed a log-linear relationship via a quasi-Poisson model. Lastly, correlation between the paired pollutants in the two-pollutant models potentially resulted in multicollinearity that produced some unstable estimates.

## Conclusions

These first contemporary population-based data from an equatorial country with tropical climate and no seasons show that exposure to particulate air pollution (PM_2.5_, PM_10_) was significantly associated with non-accidental mortality and cardiovascular mortality, especially in the elderly.

## Additional file

Additional file 1: **Table S1.** Correlation between air pollutants and meteorological data. **Table S2a.** Single-day lag two-pollutant models for association between PM_10_ and mortality. **Table S2b.** Single-day lag two-pollutant models for association between PM_2.5_ and mortality. **Table S2d.** Single-day lag two-pollutant models for association between O_3_ and mortality. **Figure S1.** Cumulative percent change (%) in non-accidental mortality for 10 μg/m^3^ pollutant concentration increase in PM_10_ (top row) and PM_2.5_ (bottom row) using 2nd degree polynomial DLM. **Figure S2.** Cumulative percent change (%) in cardiovascular mortality for 10 μg/m^3^ pollutant concentration increase in PM_10_ (top row) and PM_2.5_ (bottom row) using 2nd degree polynomial DLM. **Figure S3.** Cumulative percent change (%) in non-accidental mortality for 10 μg/m^3^ pollutant concentration increase in PM_10_ (top row) and PM_2.5_ (bottom row) using 4th degree polynomial DLM. **Figure S4.** Cumulative percent change (%) in cardiovascular mortality for 10 μg/m^3^ pollutant concentration increase in PM_10_ (top row) and PM_2.5_ (bottom row) using 4th degree polynomial DLM. **Figure S5.** Residual autocorrelation and partial autocorrelation charts for the core models. **Figure S6.** Non-cumulative percent change (%) in non-accidental mortality for 10μg/m^3^ pollutant concentration increase in PM_10_ (top row) and PM_2.5_ (bottom row). **Figure S7**. Non-cumulative percent change (%) in cardiovascular mortality for 10μg/m^3^ pollutant concentration increase in PM_10_ (top row) and PM_2.5_ (bottom row). (DOCX 322 kb)

## Abbreviations

APHEA: Air Pollution on Health: European Approach
CI: Confidence interval
CO: Carbon monoxide
DLM: Distributed lag model
ER: Excess risk
ICD: International Classification of Diseases
NEA: National Environment Agency
NO_2_: Nitrogen dioxide
O_3_: Ozone
PM_10_: Particulate matters < 10 μm
PM_2.5_: Particulate matters < 2.5 μm
RR: Relative risk
SO_2_: Sulphur dioxide
