# Supplementary material for: Particulate air pollution on cardiovascular mortality in the tropics: impact on the elderly
Source: Environ Health. 2019 Apr 18;18:34. doi: 10.1186/s12940-019-0476-4 (PMC6471752; doi:10.1186/s12940-019-0476-4)
Supplement: Supplementary file 1 — Table S1. Correlation between air pollutants and meteorological data. Table S2a. Single-day lag two-pollutant models for association between PM10 and mortality. Table S2b. Single-day lag two-pollutant models for association between PM2.5 and mortality. Table S2d. Single-day lag two-pollutant models for association between O3 and mortality. Figure S1. Cumulative percent change (%) in non-accidental mortality for 10 μg/m3 pollutant concentration increase in PM10 (top row) and PM2.5 (bottom row) using 2nd degree polynomial DLM. Figure S2. Cumulative percent change (%) in cardiovascular mortality for 10 μg/m3 pollutant concentration increase in PM10 (top row) and PM2.5 (bottom row) using 2nd degree polynomial DLM. Figure S3. Cumulative percent change (%) in non-accidental mortality for 10 μg/m3 pollutant concentration increase in PM10 (top row) and PM2.5 (bottom row) using 4th degree polynomial DLM. Figure S4. Cumulative percent change (%) in cardiovascular mortality for 10 μg/m3 pollutant concentration increase in PM10 (top row) and PM2.5 (bottom row) using 4th degree polynomial DLM. Figure S5. Residual autocorrelation and partial autocorrelation charts for the core models. Figure S6. Non-cumulative percent change (%) in non-accidental mortality for 10μg/m3 pollutant concentration increase in PM10 (top row) and PM2.5 (bottom row). Figure S7. Non-cumulative percent change (%) in cardiovascular mortality for 10μg/m3 pollutant concentration increase in PM10 (top row) and PM2.5 (bottom row). (DOCX 322 kb) [file 12940_2019_476_MOESM1_ESM.docx]

**Additional file 1**

Table S1. Correlation between air pollutants and meteorological data

|  | **PM10 (µg/m^3^)** | **PM2.5 (µg/m^3^)** | **CO (mg/m^3^)** | **NO2 (µg/m^3^)** | **O3 (µg/m^3^)** | **SO2 (µg/m^3^)** | **DBT (˚C)** | **RH (%)** |
| --- | --- | --- | --- | --- | --- | --- | --- | --- |
| **PM_10_ (µg/m^3^)** | 1.000 | 0.923 | 0.404 | 0.500 | 0.428 | 0.278 | 0.246 | -0.210 |
| **PM_2.5_ (µg/m^3^)** | - | 1.000 | 0.439 | 0.614 | 0.375 | 0.417 | 0.268 | -0.149 |
| **CO (mg/m^3^)** | - | - | 1.000 | 0.470 | 0.114 | 0.343 | -0.069 | 0.168 |
| **NO_2_ (µg/m^3^)** | - | - | - | 1.000 | 0.125 | 0.555 | 0.008 | 0.226 |
| **O_3_ (µg/m^3^)** | - | - | - | - | 1.000 | -0.033 | 0.385 | -0.485 |
| **SO_2_ (µg/m^3^)** | - | - | - | - | - | 1.000 | 0.238 | -0.110 |
| **DBT (˚C)** | - | - | - | - | - | - | 1.000 | -0.785 |
| **RH (%)** | - | - | - | - | - | - | - | 1.000 |

Table S2a: Single-day lag two-pollutant models for association between PM_10_ and mortality*

|  | **Percentage change ( 95% confidence interval)** | | | |
| --- | --- | --- | --- | --- |
|  | **Lag 0** | **Lag 1** | **Lag 2** | **Lag 3** |
| **Non Accidental Mortality** | | | | |
| **Adjusted for**  **CO** | **0.431 (0.020, 0.844)** | **0.457 (0.011, 0.904)** | **0.587 (0.024, 1.154)** | **0.670 (0.225, 1.117)** |
| **NO2** | **0.595 (0.189, 1.002)** | **0.530 (0.146, 0.914)** | **0.499 (0.125, 0.874)** | **0.636 (0.268, 1.006)** |
| **O3** | **0.426 (0.041, 0.813)** | **0.404 (0.008, 0.803)** | **0.421 (0.041, 0.802)** | **0.583 (0.210, 0.957)** |
| **SO2** | **0.478 (0.101, 0.856)** | **0.472 (0.098, 0.847)** | **0.451 (0.078, 0.825)** | **0.611 (0.243, 0.98)** |
| **Cardiovascular Mortality** | | | | |
| **Adjusted for**  **CO** | 0.564 (-0.115, 1.247) | **0.738 (0.000, 1.482)** | **0.993 (0.090, 1.904)** | **1.136 (0.396, 1.882)** |
| **NO2** | **0.710 (0.038, 1.387)** | **0.707 (0.065, 1.353)** | **0.646 (0.019, 1.277)** | **0.921 (0.303, 1.542)** |
| **O3** | 0.517 (-0.126, 1.165) | 0.597 (-0.068, 1.268) | 0.576 (-0.062, 1.217) | **0.875 (0.250, 1.503)** |
| **SO2** | 0.547 (-0.082, 1.181) | 0.617 (-0.011, 1.249) | 0.573 (-0.055, 1.205) | **0.882 (0.265, 1.503)** |

* Per 10µg/m^3^ increase in PM_10_.

Table S2b: Single-day lag two-pollutant models for association between PM_2.5_ and mortality*

|  | **Percentage change ( 95% confidence interval)** | | | |
| --- | --- | --- | --- | --- |
|  | **Lag 0** | **Lag 1** | **Lag 2** | **Lag 3** |
| **Non Accidental Mortality** | | | | |
| **Adjusted for**  **CO** | **0.526 (0.019, 1.035)** | 0.447 (-0.109, 1.005) | 0.532 (-0.180, 1.250) | **0.666 (0.108, 1.227)** |
| **NO2** | **0.754 (0.247, 1.264)** | **0.573 (0.096, 1.053)** | **0.527 (0.064, 0.992)** | **0.672 (0.214, 1.132)** |
| **O3** | **0.518 (0.044, 0.995)** | 0.407 (-0.083, 0.899) | 0.429 (-0.039, 0.900) | **0.606 (0.144, 1.071)** |
| **SO2** | **0.575 (0.111, 1.042)** | **0.486 (0.022, 0.952)** | 0.453 (-0.011, 0.920) | **0.632 (0.174, 1.092)** |
| **Cardiovascular Mortality** | | | | |
| **Adjusted for**  **CO** | 0.639 (-0.202, 1.487) | 0.676 (-0.250, 1.609) | 0.829 (-0.324, 1.995) | **1.091 (0.168, 2.022)** |
| **NO2** | **0.861 (0.015, 1.714)** | 0.702 (-0.101, 1.512) | 0.589 (-0.192, 1.376) | **0.914 (0.148, 1.687)** |
| **O3** | 0.589 (-0.206, 1.390) | 0.540 (-0.283, 1.371) | 0.495 (-0.295, 1.291) | **0.851 (0.077, 1.630)** |
| **SO2** | 0.614 (-0.164, 1.398) | 0.565 (-0.217, 1.354) | 0.472 (-0.315, 1.265) | **0.854 (0.087, 1.628)** |

* Per 10µg/m^3^ increase in PM_2.5_.

Table S2c: Single-day lag two-pollutant models for association between O_3_ and mortality*

|  | **Percentage change ( 95% confidence interval)** | | | |
| --- | --- | --- | --- | --- |
|  | **Lag 0** | **Lag 1** | **Lag 2** | **Lag 3** |
| **Non Accidental Mortality** | | | | |
| **Adjusted for**  **PM10** | -0.058 (-0.446, 0.332) | 0.265 (-0.082, 0.613) | -0.127 (-0.529, 0.276) | 0.027 (-0.330, 0.385) |
| **PM2.5** | -0.039 (-0.427, 0.351) | 0.285 (-0.061, 0.632) | -0.100 (-0.502, 0.303) | 0.066 (-0.290, 0.423) |
| **CO** | -0.026 (-0.415, 0.365) | 0.322 (-0.023, 0.669) | -0.045 (-0.449, 0.361) | 0.195 (-0.143, 0.533) |
| **NO2** | 0.044 (-0.383, 0.472) | **0.398 (0.041, 0.757)** | 0.053 (-0.351, 0.458) | 0.228 (-0.111, 0.569) |
| **SO2** | 0.016 (-0.371, 0.404) | 0.341 (-0.001, 0.686) | 0.012 (-0.382, 0.408) | 0.226 (-0.110, 0.563) |
| **Cardiovascular Mortality** | | | | |
| **Adjusted for**  **PM10** | 0.403 (-0.235, 1.044) | 0.166 (-0.400, 0.736) | -0.072 (-0.644, 0.504) | 0.189 (-0.489, 0.870) |
| **PM2.5** | 0.435 (-0.202, 1.076) | 0.203 (-0.362, 0.772) | -0.024 (-0.596, 0.551) | 0.253 (-0.431, 0.942) |
| **CO** | 0.485 (-0.152, 1.127) | 0.278 (-0.284, 0.844) | 0.087 (-0.477, 0.654) | 0.471 (-0.182, 1.129) |
| **NO2** | 0.662 (-0.030, 1.359) | 0.351 (-0.231, 0.935) | 0.140 (-0.431, 0.715) | 0.525 (-0.127, 1.181) |
| **SO2** | 0.508 (-0.126, 1.146) | 0.277 (-0.283, 0.840) | 0.070 (-0.494, 0.636) | 0.487 (-0.158, 1.135) |

* Per 10µg/m^3^ increase in O_3_.

Figure S1. Cumulative percent change (%) in non-accidental mortality for 10µg/m^3^ pollutant concentration increase in PM_10_ (top row) and PM_2.5_ (bottom row) using 2^nd^ degree polynomial DLM.


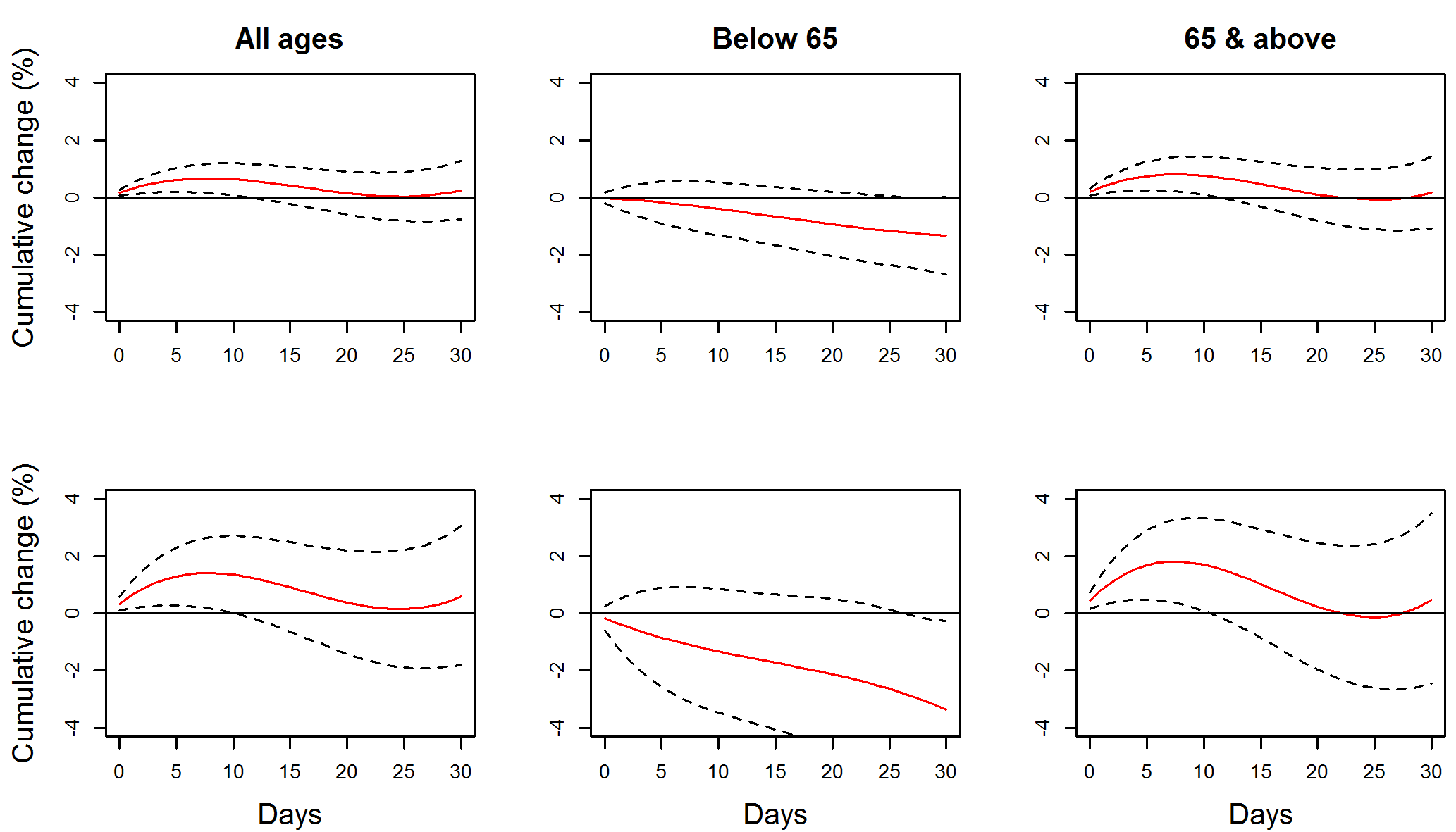


Figure S2. Cumulative percent change (%) in cardiovascular mortality for 10µg/m^3^ pollutant concentration increase in PM_10_ (top row) and PM_2.5_ (bottom row) using 2^nd^ degree polynomial DLM.


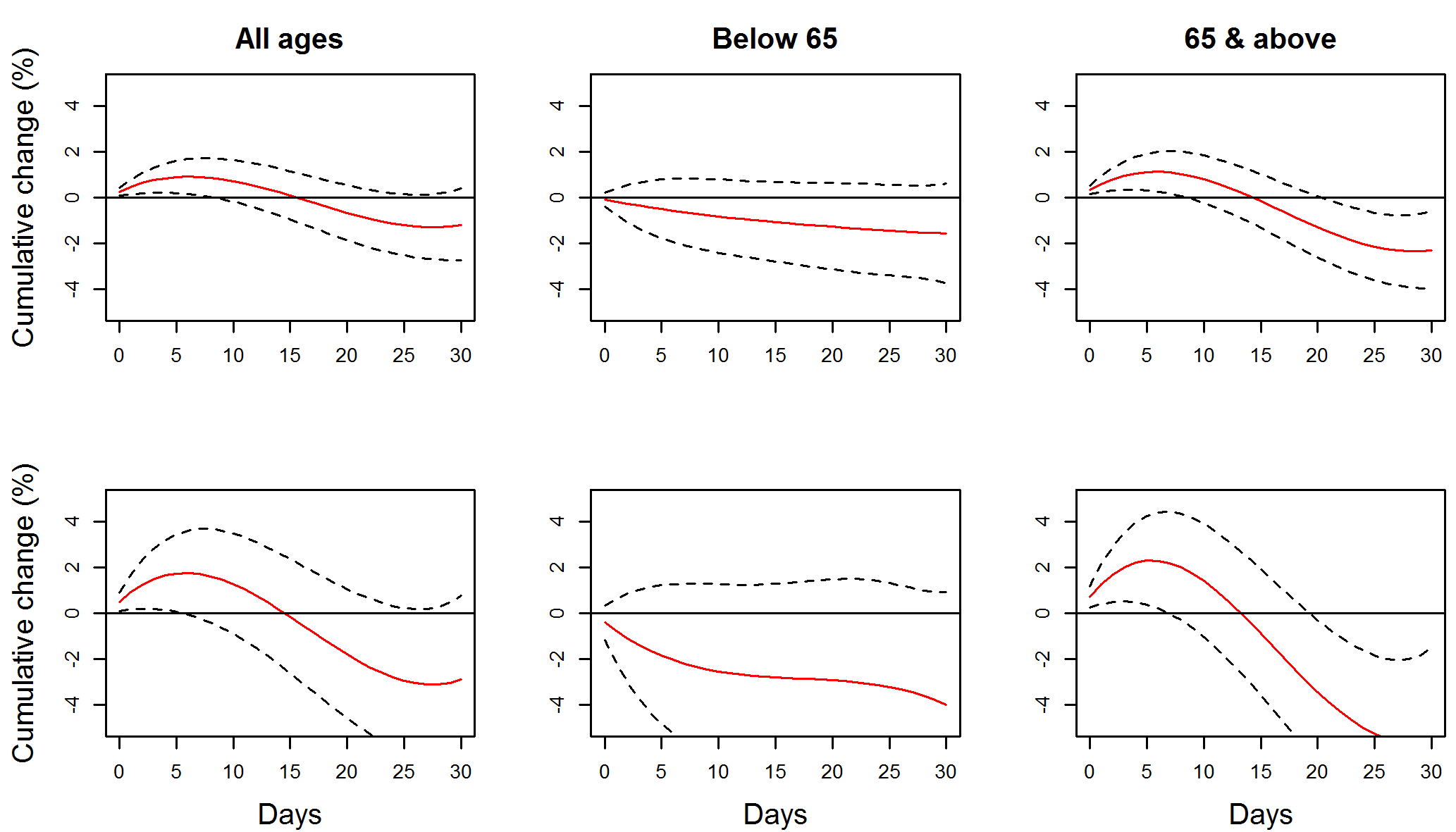


Figure S3. Cumulative percent change (%) in non-accidental mortality for 10µg/m^3^ pollutant concentration increase in PM_10_ (top row) and PM_2.5_ (bottom row) using 4^th^ degree polynomial DLM.


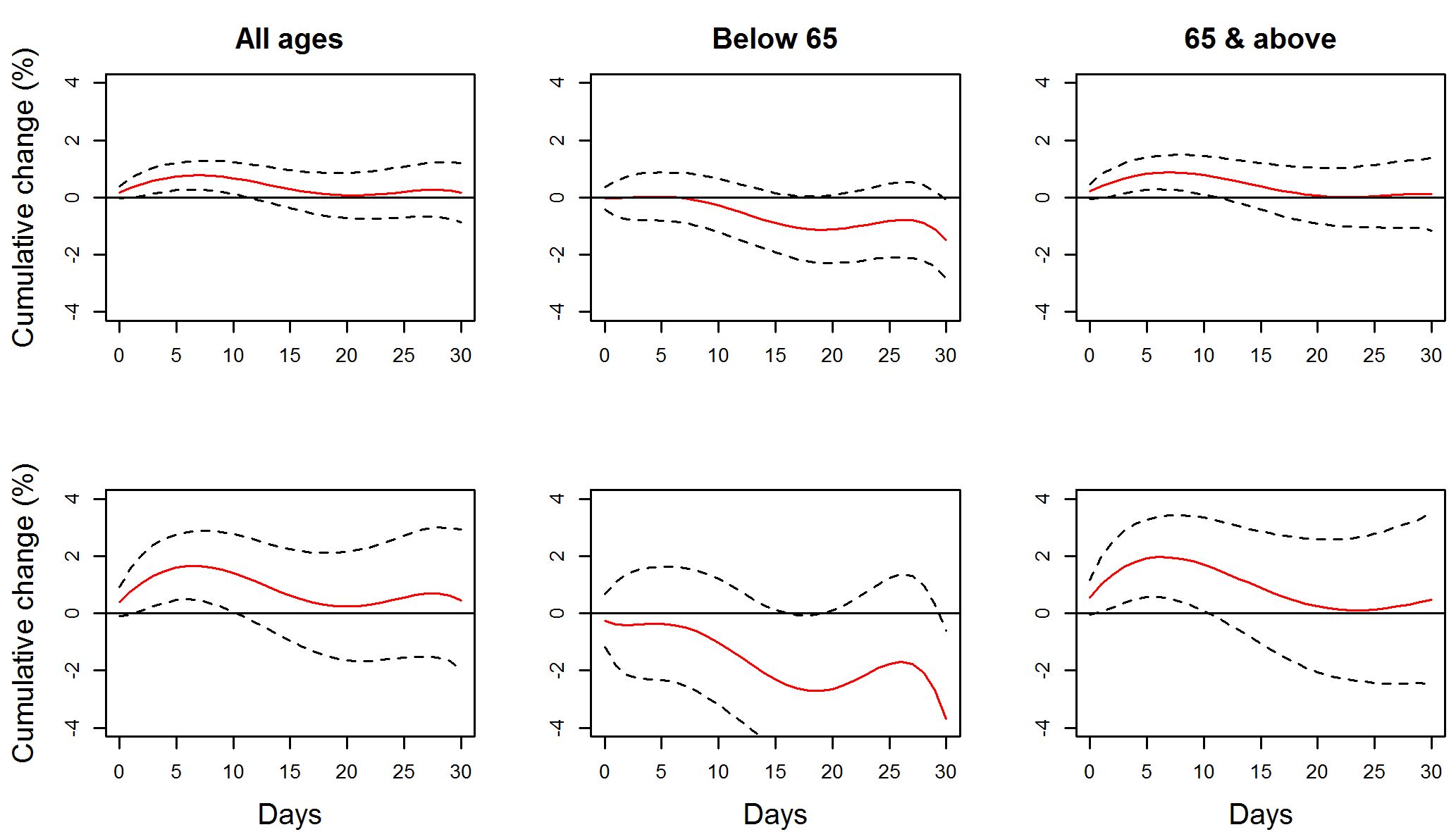


Figure S4. Cumulative percent change (%) in cardiovascular mortality for 10µg/m^3^ pollutant concentration increase in PM_10_ (top row) and PM_2.5_ (bottom row) using 4^th^ degree polynomial DLM.


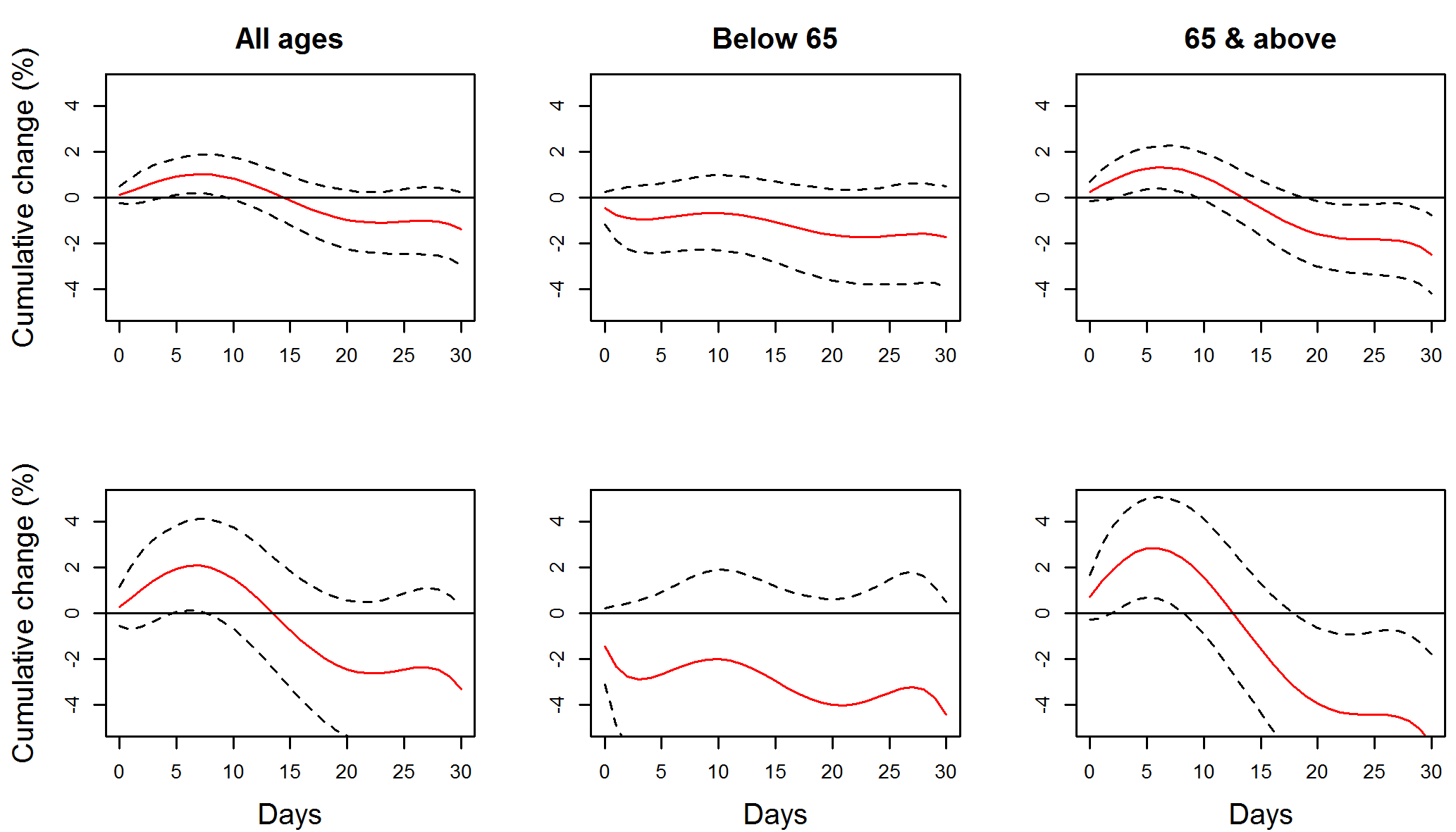


Figure S5. Residual autocorrelation and partial autocorrelation charts for the core models.


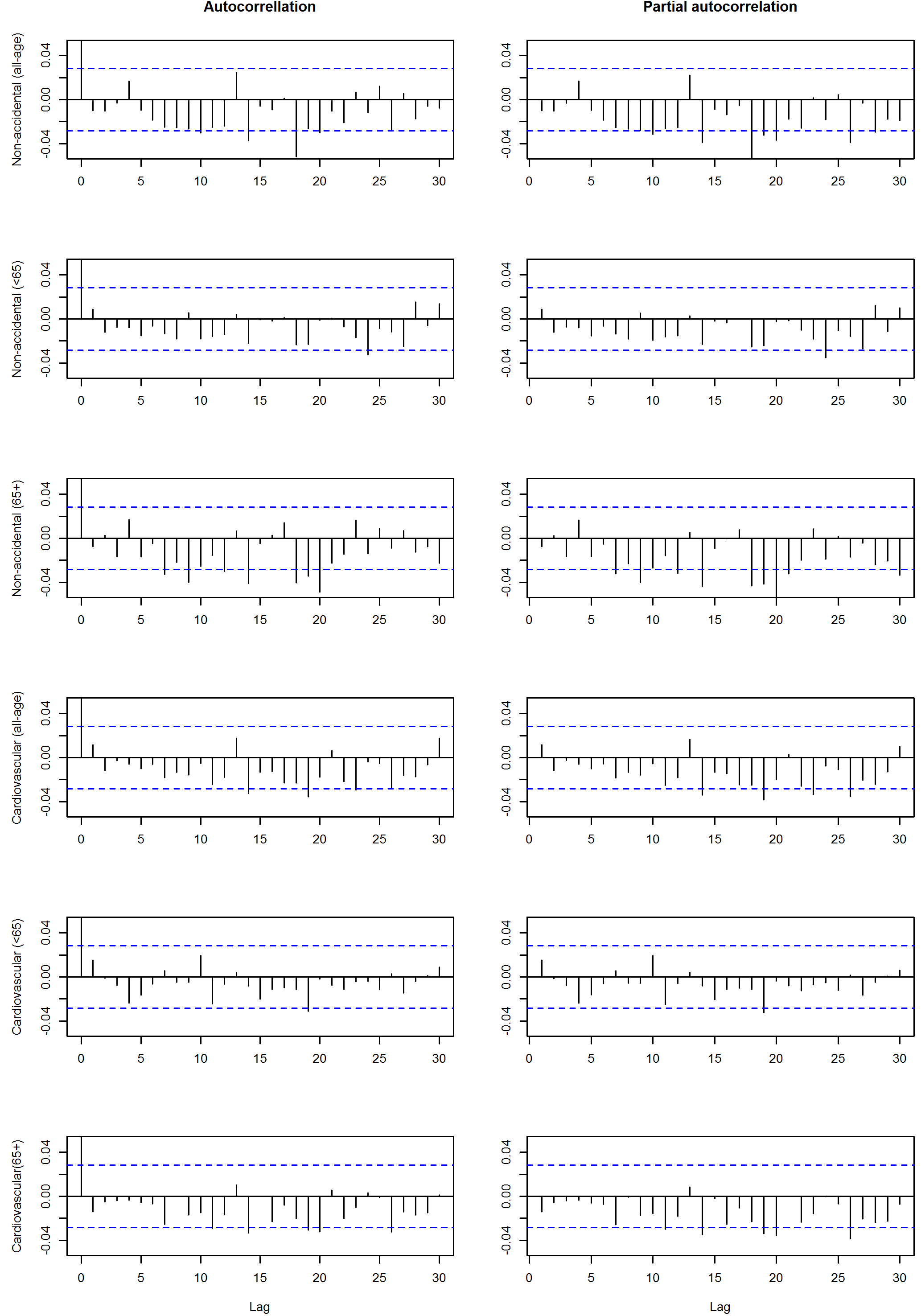


Figure S6. Non-cumulative percent change (%) in non-accidental mortality for 10ug/m^3^ pollutant concentration increase in PM_10_ (top row) and PM_2.5_ (bottom row).


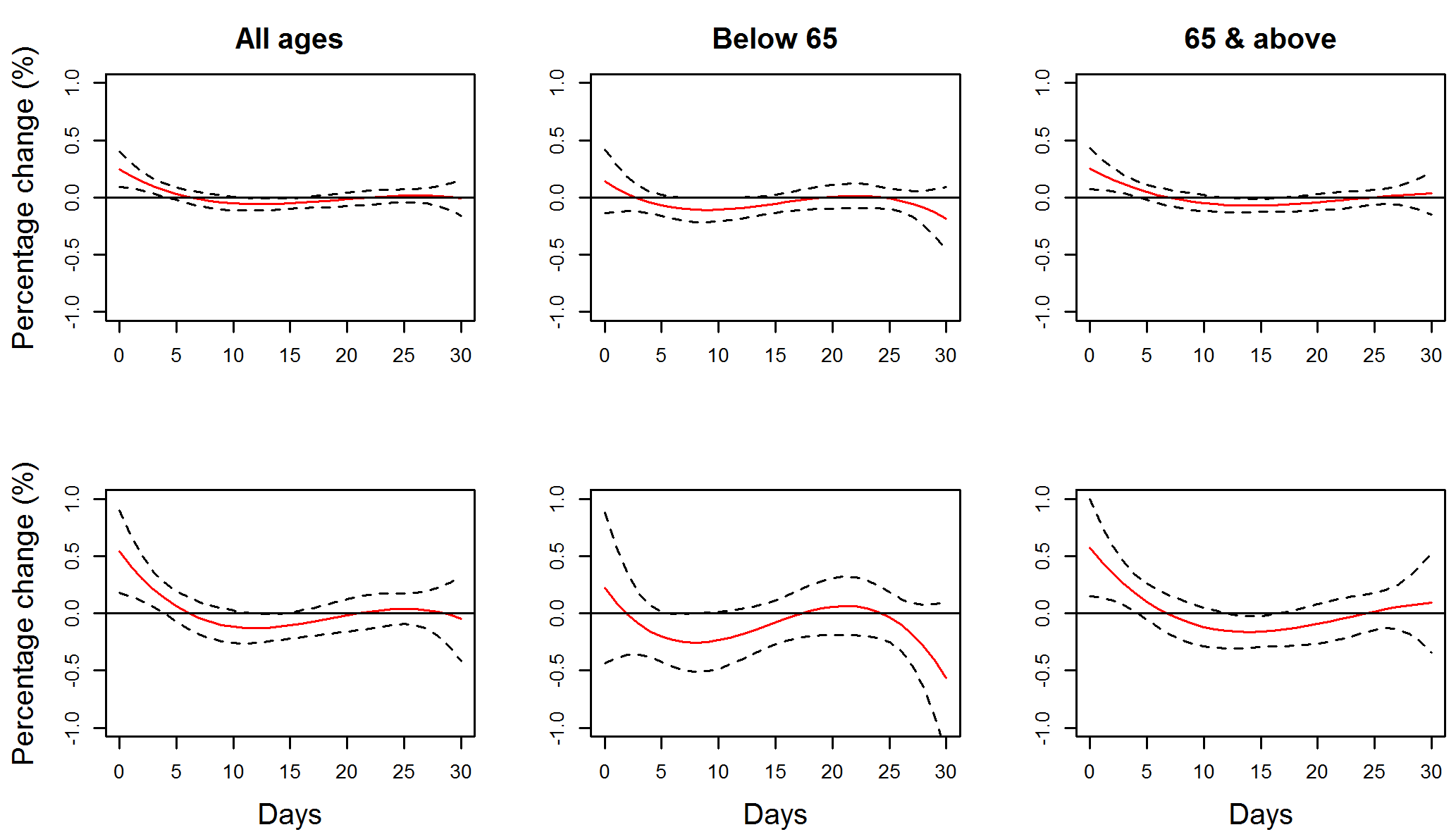


Figure S7. Non-cumulative percent change (%) in cardiovascular mortality for 10ug/m^3^ pollutant concentration increase in PM_10_ (top row) and PM_2.5_ (bottom row).

**
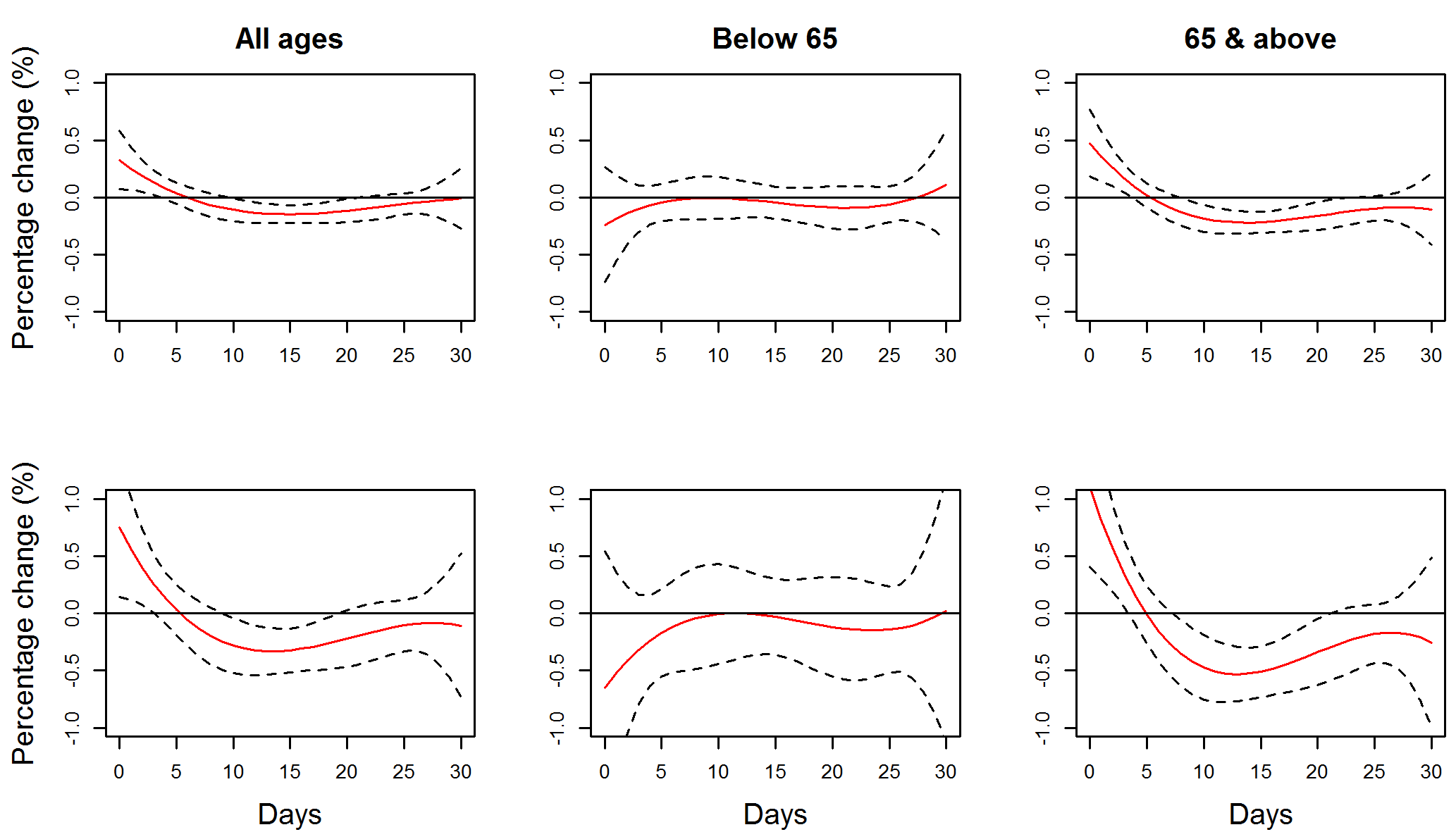
**
